# Assessment of Attentional Functioning in Health Professionals of a Brazilian Tertiary Referral Hospital for COVID-19

**DOI:** 10.1155/2021/6655103

**Published:** 2021-06-25

**Authors:** Eelco van Duinkerken, Guilherme J. Schmidt, Ana Lúcia Taboada Gjorup, Carolina Ribeiro Mello, André Casarsa Marques, Áureo do Carmo Filho, Paula Regina Yuri Fukusawa, Simone Gonçalves de Assis, Júlio Cesar Tolentino, Sergio L. Schmidt

**Affiliations:** ^1^Department of Neurology, University Hospital Gaffrée and Guinle, Federal University of the State of Rio de Janeiro, Rio de Janeiro, RJ, Brazil; ^2^Department of Medical Psychology, Amsterdam University Medical Centers, Vrije Universiteit, Amsterdam, Netherlands; ^3^Amsterdam Diabetes Center/Department of Internal Medicine, Amsterdam University Medical Centers, Vrije Universiteit, Amsterdam, Netherlands; ^4^Department of Internal Medicine, University Hospital Gaffrée and Guinle, Federal University of the State of Rio de Janeiro, Rio de Janeiro, RJ, Brazil; ^5^Department of Anesthesiology, University Hospital Gaffrée and Guinle, Federal University of the State of Rio de Janeiro, Rio de Janeiro, RJ, Brazil; ^6^Education and Research Unit, University Hospital Gaffrée and Guinle, EBSERH, Federal Ministry of Education and Culture, Brazil; ^7^Fundação Oswaldo Cruz, Rio de Janeiro, RJ, Brazil

## Abstract

This study is aimed at assessing differences in basic attentional functioning between substantial and minimal work-related exposure to COVID-19 patients in professionals working in a tertiary referral hospital in Rio de Janeiro, Brazil. Therefore, hospital employees performed a Continuous Visual Attention Test. This test consisted of a 90-second Go/No-Go task with 72 (80%) targets and 18 (20%) nontargets. For each participant, reaction time and intraindividual variability of reaction times of all correct target responses, as well as the number of omission and commission errors, were evaluated. Participants were divided into 2 groups based on their exposure to COVID-19 patients (substantial versus minimal exposure). The substantial exposure group consisted of participants with 24 hours/week or more direct contact with COVID-19 patients. This cut-off was based on the clear division between professionals working and not working with COVID-19 patients and considered that 12-hour and 24-hour daily shifts are common for hospital employees in Brazil. A MANCOVA was performed to examine between-group differences, using age, sleep quality, sex, education level, previous COVID-19 infection, and profession as covariates. Of 124 participants, 80 had substantial exposure and 44 had minimal exposure to COVID-19. The overall MANCOVA reached statistical significance (*P* = 0.048). Post hoc ANCOVA analysis showed that the substantial exposure group had a statistically significantly higher intraindividual variability of reaction time of all correct target responses (*P* = 0.017, Cohen′s *δ* = −0.55). This result remained after removing those with a previous COVID-19 infection (*P* = 0.010, Cohen′s *δ* = −0.64) and after matching groups for sample size (*P* = 0.004, Cohen′s *δ* = −0.81). No other variables reached statistical significance. Concluding, hospital professionals with a substantial level of exposure to patients with COVID-19 show a significant attention decrement and, thus, may be at a higher risk of accidental SARS-CoV-2 infection.

## 1. Introduction

Since the outbreak of the COVID-19 pandemic, health care professionals are at a high risk to be infected by the SARS-CoV-2 virus and are prone to develop psychological problems. Indeed, a large study in more than 1,200 health care professionals from 34 different hospitals in China has shown that symptoms of depression, anxiety, stress, and insomnia were particularly prevalent [1]. Thus, it is important to identify the psychological burden of professionals working in tertiary referral hospitals for COVID-19, including not only physicians and nurses, but also laboratory technicians, psychologists, physiotherapists, security, and administrative personnel.

There could be numerous reasons why health care professionals may experience adverse psychological effects during this pandemic, including little or no training to assist patients with anxiety, panic, and other emotional problems, a lack of sufficient protective equipment, increasing work load and concomitant fatigue, and feelings of inadequacy in treating critically ill patients [2, 3]. Previous studies not related to COVID-19 have indeed shown that long working hours under stressful conditions increase the risks of mental health complications [4]. Thus, another factor that could impact psychological well-being of health care personnel is the total exposure time, that is, how many hours a week they directly work with COVID-19 patients. The amount of time workers spends doing activities with COVID-19 patients may increase risk of infection and psychological burden. However, the effect of exposure time to COVID-19 on psychological health is not known.

Besides psychological distress and burden, health care professionals may also be faced with (transient) cognitive effects, which may be related to the aforementioned psychological symptoms. Whether these possible alterations improve with improving psychological well-being, either naturally or via therapy, is unknown. However, experiencing cognitive problems can directly affect work performance, worsen feelings of distress, depression, and anxiety, and increase worrying [5, 6], which could ultimately lead to a vicious cycle and burnout.

Attention is the ability to choose and concentrate on relevant stimuli and is considered a core human cognitive function [7, 8]. Thus, a decrease in attentional performance could lead to a higher risk of accidental SARS-CoV-2 infection, as workers might be more prone to make mistakes. Despite the importance of attention performance for cognitive functioning and work safety, we have not found studies on attention assessment in health care professionals working with COVID-19 patients.

Basic attention can be reliably measured by simple reaction time or Go/No-Go tasks, which have commonly the advantage of relying less on intelligence and education level than more complicated neuropsychological tests [7]. One such Go/No-Go test, the Continuous Visual Attention Test (CVAT), has been shown to be capable to detect attentional alterations in various conditions, including ADHD [9], chronic pain [10], and in people with obstructive sleep apnea [11]. Besides reaction time and errors, this test produces intraindividual variability of reaction time. This measure can be considered as the fluctuation in reaction time during test performance and as such a lower variability indicates a more stable performance in terms of reaction time. Moreover, variability is considered a prime measure and has been linked to various attentional, including sustained attention [11], cognitive [12], and psychological processes [13].

Considering the importance of attentional functioning for work and mental well-being, we set out to measure attention in health care professionals with substantial and minimal exposure time to COVID-19 working in a COVID-19 tertiary referral hospital in Rio de Janeiro, Brazil. In this study, we determined the effect of exposure time to COVID-19 patients on attentional functioning, hypothesizing that the group with substantial exposure time would have worse functioning as compared to the group with minimal exposure time.

## 2. Materials and Methods

### 2.1. Participants

This study was approved by the Medical Ethics Committee of the University Hospital Gaffrée and Guinle and by the federal Medical Ethics Committee (CAAE: 30547720.3.0000.0008). The study was conducted in accordance with the Declaration of Helsinki, and written informed consent was obtained from all participants. Inclusion criteria for this study were an age between 20 and 60 years and being a hospital employee. Exclusion criteria for this study included conditions that may interfere with attentional performance, such as any previous or current neurological disorder or diabetes and the use of benzodiazepines, antidepressants, all hypnotic sedatives, all antipsychotics, antiallergics that cross the blood brain barrier, glucocorticoids, or all muscle relaxants. Additionally, participants were excluded when they had less than 50% correct responses on the CVAT task, which indicates a performance below chance level, or when they performed the test at the end of their work day, to control for potential effects of fatigue on performance.

### 2.2. Exposure to COVID-19 Patients

Every participant was asked about his/her recent exposure to COVID-19 patients in the past week. Two groups were created one with substantial and one with minimal exposure to COVID-19 patients, with a cut-off of 24 hours of exposure. This cut-off was chosen as in Brazil 12-hour and 24-hour daily shifts are common for medical employees, psychologists, physiotherapists, and laboratory professionals working in hospitals, and contractual working hours consisted of 24 or 40 hours a week. Furthermore, in this tertiary hospital, there was a clear division between professionals working and not working with COVID-19 patients. Thus, participants had either no to minimal exposure or substantial exposure to COVID-19 as can also be found in Table 1.

### 2.3. Attentional Functioning

**Figure 1:**
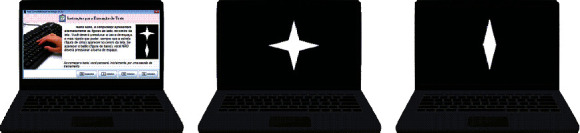
Schematic overview of the set-up of the laptop and of the target (star) and nontarget (diamond).

For this study, a 90-second computerized Go/No-Go test was used (Figure 1). The target stimulus consisted of a star presented in the middle of the screen, whereas the nontarget stimulus was a diamond. The CVAT consisted of one block of 90 trials, each trial being presented for 250 ms, with an interstimulus interval of 750 milliseconds and a stimulus onset asynchrony of 1 second. Of the 90 trials, 72 (80%) were targets (stars), and 18 (20%) were nontargets (diamonds).

Subjects were seated in front of the computer in such a way as to allow the hands to be comfortably placed over the keyboard, and that the distance to the center of the monitor was approximately 50 cm. Instructions were shown on the screen and then reinforced by the examiner. A practice session, in which no errors could be made, was presented before testing commenced. In the case of errors, the practice session was automatically repeated.

For each participant, the mean reaction time of correct responses to the target was calculated. Additionally, as reaction times will vary throughout the test, intraindividual variability of all reaction times of the correct responses to the target was also determined. Here, each individual's variability was calculated by using the standard deviation of the individual distribution of all reaction times of the correct responses to the target. Intraindividual variability of reaction times may depend on reaction time itself. This indicates that, in case of a difference in reaction time, possible alterations in intraindividual variability might be influenced by reaction time itself [14, 15]. To circumvent this potential bias, we calculated the coefficient of intraindividual variability, which is calculated as the intraindividual variability divided by reaction time. Lastly, omission errors (no response to a target) and commission errors (response to a nontarget) were calculated.

Fast responses, those faster than 150 ms, are most likely physiologically implausible. Therefore, those fast responses were removed, and for those participants, the mean reaction time, intraindividual variability of all reaction times, and the coefficient of variability were recalculated.

### 2.4. Statistical Analyses

Demographic variables were analyzed using an independent sample *t*-test for normally distributed variables, a Kruskal-Wallis test for nonnormally distributed variables, or a *χ*^2^ test for categorical variables. Normality of variables was confirmed by assessing the histograms, boxplots, and QQ plots. Effect sizes were calculated as Cohen's *δ*, where a *δ* = 0.20 is considered a small effect, a *δ* = 0.50 a medium effect, and *δ* = 0.80 or higher a large effect [16].

First, a MANCOVA was performed including reaction time, variability of reaction time, omission and commission errors as dependent variables, and exposure to COVID-19 (minimal or substantial) as independent variable. This analysis was corrected for age, sex, sleep quality, education level, previous COVID-19 infection, and profession. Sleep quality was defined by asking the participant to rate the quality of their sleep in the past 2 weeks. Box's M test was used to assess the homogeneity of the covariance matrices. In case of a significant overall MANCOVA, the post hoc ANCOVA of each of the dependent variables was checked for statistical significance. A significant MANCOVA indicates that at least one dependent variable is different between the groups, thus allowing for further post hoc testing. A MANCOVA/ANCOVA approach was chosen as it has been shown to give robust results even when variables are not normally distributed [17].

A *P* < 0.05 was considered to be statistically significant. All analyses were performed using SPSS version 26 (IBM-SPSS, Chicago, IL, USA).

## 3. Results

### 3.1. Participants

From the start of the study, mid-May, until July 1^st^ 2020, a total of 161 hospital employees were included into the study. After applying the abovementioned exclusion criteria, 21 participants were excluded. Additionally, two participants were excluded because they had a performance of less than 50% rightly signaled targets, 13 participants could not inform their exposure time to COVID-19, and 1 participant did not answer the question about sleep. Of the 124 eligible participants, 80 had substantial and 44 had a minimal exposure to COVID-19. As can be found in Table 1, those in the substantial exposure group had more often a lower level of education, and 11 did not have a bachelor's degree compared to none in the minimal exposure group (*P* = 0.014). Obviously, more participants in the substantial exposure to COVID-19 group had contracted a COVID-19 infection (22.5 versus 9.1%), which was borderline significant (*P* = 0.085). There were no other noteworthy between-group differences.

### 3.2. Variability of Reaction Times Was Higher in the Substantial Exposure Group

Evaluation of normality showed that reaction time of correct responses and the intraindividual variability of reaction times of correct responses had a normal distribution. Commission and omission errors did not, but as a MANCOVA/ANCOVA approach is robust against violation of normality [17], they can be included in the model. The analyses were corrected for age, sex, sleep quality, education level, previous COVID-19 infection, and profession.

**Figure 2:**
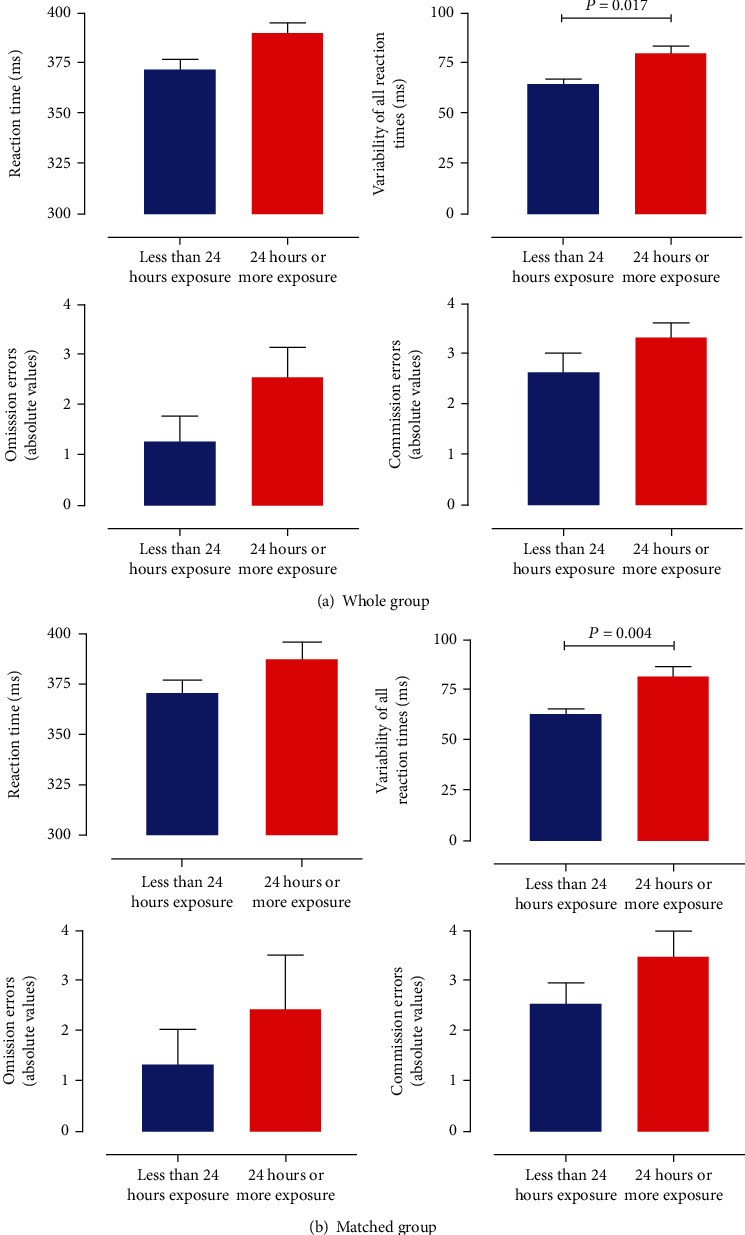
(a) Bar graphs of the mean reaction time of the correct responses, mean variability of all reaction times of the correct responses, and mean omission and commission errors with standard error of the mean of the analysis with all participants included. (b) Bar graphs of the mean reaction time of the correct responses, mean variability of all reaction times of the correct responses, and mean omission and commission errors with standard error of the mean of the analysis of both groups matched for size. Blue bars depict participants with minimal COVID-19 exposure, and red bars represent those with substantial exposure to COVID-19 patients.

**Figure 3:**
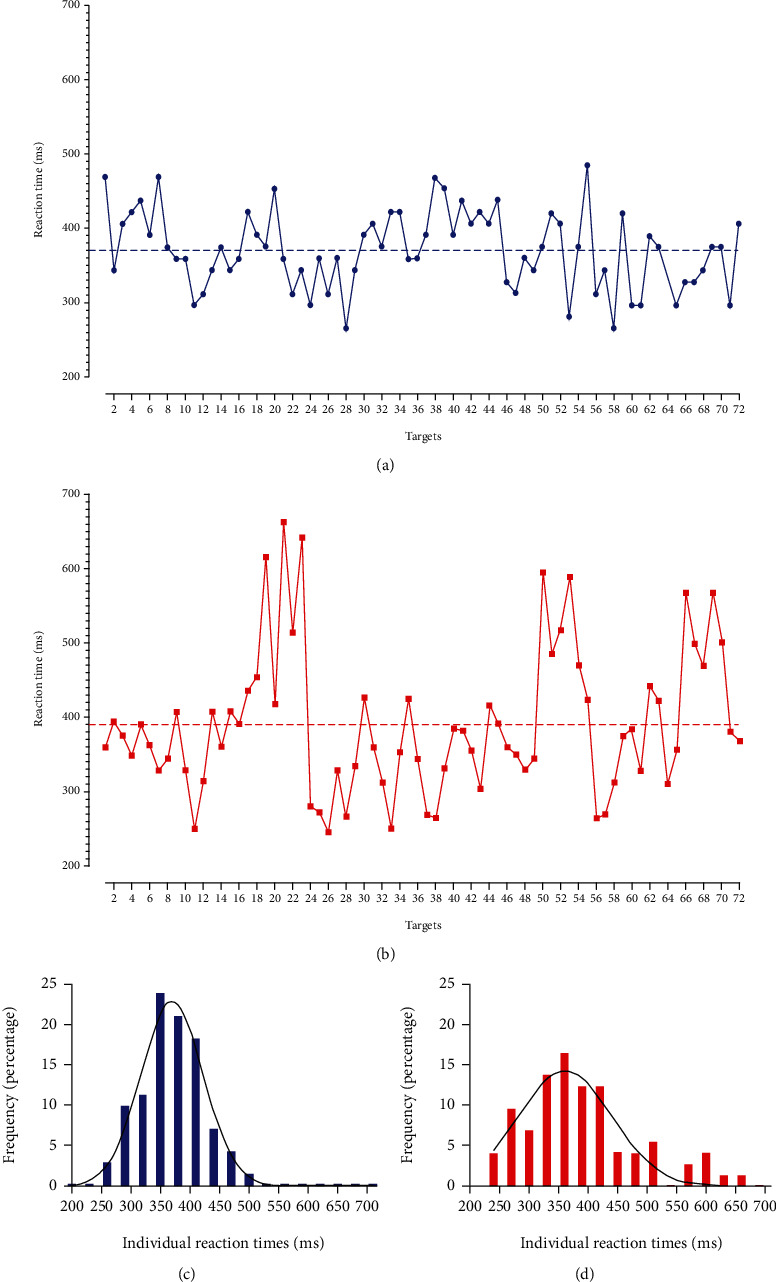
Graph of two typical participants, one in the minimal exposure group (blue circles), and one in the substantial exposure group (red squares). Both have a similar mean reaction time as represented by the dashed lines, but their variability of reaction times was different (blue: 52 ms versus red: 96 ms). Below, a histogram of all reaction times with Gaussian curve is plotted, clearly showing a wider and lower curve for the participant in the 24 hours or more exposure group, indicative of larger variability in reaction times.

Figure 2(a) shows the mean reaction time, variability of reaction times, and commission and omission errors (Figure 2 and Table 1). The overall MANCOVA reached statistical significance (*F* = 2.48, df: 4/116, and *P* = 0.048). The post hoc ANCOVAs showed that intraindividual variability of correct response times was statistically significantly higher in the substantial exposure to COVID-19 group than in the minimal exposure group (*F* = 5.83, df: 1/116, *P* = 0.017, and Cohen′s *δ* = −0.55). Although reaction time (*P* = 0.075), the number of omission (*P* =0.126) and the number of commission (*P* =0.229) errors were also higher in the substantial versus minimal exposure group, none were statistically significant. To illustrate intraindividual variability of correct response times, we selected 2 participants (one from each group) and plotted their reaction times in Figure 3. The participant from the minimal exposure group (blue) had a mean reaction time of 370 ms and a mean intraindividual variability of correct response times of 52 ms, whereas the participant from the substantial exposure group (red) had a mean reaction time of 390 ms with an intraindividual variability of correct response times of 96 ms. As can be seen in Figure 3, for a lower mean intraindividual variability of correct response times, the individual reaction times tend to center more around the mean, whereas for a high intraindividual variability of correct response times, the reaction times show a larger spread.

As the higher reaction time in the group with substantial exposure to COVID-19 approached statistical significance, it may partly explain the findings of variability of the reaction times. Therefore, we analyzed the difference in the coefficient of variability of reaction times, which eliminates the influence of reaction time on variability of reaction times [14, 15]. As can be found in Table 1, the coefficient of variability was higher in the substantial exposure group (*F* = 4.23, df: 1/116, *P* = 0.042, and Cohen′s *δ* = 0.56). This demonstrates that the statistically significant difference found for variability of reaction times was not driven by a difference in reaction times itself.

### 3.3. Previous COVID-19 Infection Did Not Affect Performance

An infection with COVID-19 may affect cognitive and attentional performance. Therefore, we have used this variable as a confounding factor. Additionally, we repeated the MANCOVA removing the 22 participants (4 in the minimal and 18 in the substantial exposure group) from the analyses. Despite the loss of degrees of freedom and potential statistical power, the overall MANCOVA remained statistically significant (*F* = 3.40, df: 4/95, and *P* = 0.012), with variability of reaction times (*F* = 6.88, df: 1/95, *P* = 0.010, and Cohen′s *δ* = 0.62) and the coefficient of variability (*F* = 5.67, df: 1/95, *P* = 0.019, and Cohen′s *δ* = 0.55), being the only variables reaching statistical significance. This indicates that a previous infection of COVID-19 did not have a major effect on the here presented results.

### 3.4. Variability of Reaction Times Remains Higher after Matching Groups for Size

An imbalance in sample size has been found to be able to negatively affect ANCOVA results [18]. Therefore, as an additional analysis, we performed one-on-one matching based on age and sex, including 32 participants in both groups and repeated the ANCOVA for variability of reaction times and the coefficient of variability, correcting for age, sex, sleep quality, education, previous COVID-19 infection, and profession.  Table 2 shows the characteristics of these groups, where no differences were noted between them (all *P* > 0.05).

Figure 2(b) shows group performance schematically in a bar graph. The ANCOVAs for both variability of reaction times (*F* = 9.06, df: 1/56, *P* = 0.004, and Cohen′s *δ* = 0.81) and the coefficient of variability (*F* = 7.52, df: 1/56, *P* = 0.008, and Cohen′s *δ* = 0.81) remained statistically significant. Excluding those with a previous COVID-19 infection did not alter the results.

## 4. Discussion

In this study, we set out to identify alterations in attentional functioning, a core cognitive function, in health care professionals with substantial and minimal exposure to COVID-19. Using a brief 90-second Go/No-Go test, we showed that the substantial exposure group exhibited lower attentional performance as compared to the minimal group. In particular, performance was less stable over the duration of the test as evidenced by a significant increase in variability of reaction times in the substantially exposed group as compared to the minimally exposed group. Reaction times and performance accuracy did not reach statistical significance. This finding was robust and not influenced by a previous COVID-19 infection or unequal group size.

Where concepts such as reaction time, commission, or omission errors are relatively straightforward, variability of reaction times may be more difficult to understand. A higher variability indicates that responses during the test show greater fluctuations in reaction times. Translated to our results, it indicates that those with substantial exposure to COVID-19 exhibited larger fluctuations in response times, which is clearly demonstrated in Figure 3. In Figures 3(a) and 3(b), 2 graphs are shown of the reaction time to each target of a participant with minimal (a) and of a participant with substantial (b) exposure to COVID-19. Whereas the dotted line showing the mean reaction time indicates that they were similar between both participants, it becomes clear that the individual reaction times fluctuate more for the participant of the substantial exposure group. For the other participant, the individual reaction times center more around the mean. Indeed, the variability was almost 2 times higher for the participant with substantial exposure than compared to the one with minimal exposure to COVID-19. This is further illustrated in Figures 3(c) and 3(d). The histogram of all reaction times of the participant with minimal exposure (blue) shows a stronger centering around the mean and a more normal distribution than the histogram of the participant with substantial exposure (red).

The clinical and psychological meaning of increased variability in reaction times could not be tested in this study. However, previous studies have demonstrated that variability of reaction times is related to various different (neuro) psychological conditions. For example, higher variability, both in younger and older subjects, was related to worse cognitive functioning in domains including memory, intelligence, and information processing speed [19]. This clearly demonstrates the importance of basic attentional functioning and in this case of variability in reaction times for higher-order cognitive functions, including memory and executive functions. In another study, reaction time was measured in 790 community-dwelling elderly (70+ years of age) using a simple reaction time test. Interestingly, results showed that variability of reaction times was strongly related to increased mortality over 17 years [12]. Moreover, variability of reaction times remained strongly associated with mortality in a full model, which also included variables such as age, dementia status, smoking, and depression [12]. Additionally, in a large sample of community-dwelling people aged 18 to 85, larger variability in reaction times was both associated with increasing age and with depression [20], and, in another study, higher variability has also been found to discriminate children with ADHD from their peers [21, 22]. Lastly, larger variability of reaction times also seems to be predictive of higher psychological distress, as evidence by results from the UK Health and Lifestyle Survey [13]. Taken together, it is clear that variability of reaction times has clear psychological, neuropsychological, and clinical importance, and future studies should investigate the longitudinal importance of variability in professionals with different levels of exposure to COVID-19.

The cerebral underpinnings of variability of reaction times have been studied less extensively than those of reaction time itself. In a group of 87 healthy students, variability was linked to EEG recordings and in particular to the P3b event-related potential [23]. The P3b is a subcomponent of the P300 event-related potential and is strongest in the parietal areas, arising around 300 ms after an event [23]. In those students with high variability in reaction times, the latency of the P3b was increased with reduced amplitude, suggesting variability is a measure of noise in neural processing [23]. A functional MRI study using a Go/No-Go task in healthy volunteers showed that increased variability of reaction times was related to an increased response in a network comprising the bilateral middle frontal and right inferior frontal, parietal, and thalamus regions [24]. These regions, falling within the frontoparietal cognitive control network, have been found in other studies as well [25]. This network has been linked to cognitive control of behavior and monitoring of the external environment [26, 27]. Although increased activation in relation to increased variability may seem counterintuitive at first glance, it may suggest that greater top-down executive control is necessary in the case of higher variability [24]. The cerebral correlates could not be studied in the current preliminary assessment, but should be included to evaluate structural and functional cerebral involvement in reaction time performance in health care professionals exposed to COVID-19.

Interestingly, the increase in variability of reaction times was not accompanied by an increase in omission or commission errors, which indicates that the lapses in attention were not mirrored by lower accuracy. One can speculate that our group with substantial exposure to COVID-19 was capable of keeping up performance on a basic and easy to perform Go/No-Go task. Whether this holds true for, for example, work-related functions could not be determined in this study. Alternatively, because of the short duration of the test, the possibility of commission errors was with 20% relatively low, compared to an 80% chance of making omission errors. This methodological difference may have prevented us from finding a higher error rate in the substantial exposure to COVID-19 group. Future studies using longer versions of Go/No-Go tasks need to be used to determine the effect of exposure to COVID-19 on accuracy.

Some studies seem to indicate that having had a COVID-19 infection can negatively influence cognitive performance [28], although more studies are needed. As the number of previous COVID-19 infections is nonsignificantly higher in the group substantial exposure to COVID-19, this could in part explain our findings. Therefore, we have used previous COVID-19 infection as a confounding factor, which did not alter the results. In a secondary analysis, we excluded the participants with a previous COVID-19 infection and reran the analyses. Again, variability of reaction times remained statistically significantly higher in the substantial exposure to COVID-19 group relative to the minimal exposure group. This indicates that our results may not be driven by a previous COVID-19 infection.

In the current study, intraindividual variability of reaction time was defined as the standard deviation of all individual reaction times associated with correct responses to the target. Previous investigations using reaction-time tasks have suggested that these distributions can also be fitted by ex-Gaussian distributions [29]. Ex-Gaussian distributions are described by the convolution of a normal distribution and an additional exponential function [30]. This gives three independent parameters, mu that represents the mean of the normal component, sigma, which corresponds to the standard deviation of the normal component, and tau that corresponds to the variability of the exponential function [31]. However, our data demonstrate that the sample means are independent of the standard deviations, which gives support for the assumption of a Gaussian distribution of the reaction time data. In addition, a previous study, comparing multiple measures of variability, including the standard deviation, found that all produced similar results and were related to clinical features [32].

The number of targets and nontargets determines the reliability of measures of reaction time, variability of reaction time, and accuracy. The here used test consisted of 72 targets and 18 nontargets. Previous studies have shown that both reaction time and variability of reaction time can be reliably measured by tests as short as 52 seconds with 20 items [29, 33, 34]. In that regard, the here used 90-second CVAT has sufficient targets to reliably measure reaction time and variability of reaction time. Regarding accuracy, with 72 targets, the possibility for omission errors is relatively high. This, however, is at the expense of commission errors, as the possibility of false positive responses is limited to 18. Consequently, the use of the 90-second CVAT may have decreased the possibility to capture accuracy alterations.

A commonly used measure related to accuracy is *d*′, which represents the sum of the normalized commission and omission errors. To calculate this measure, it is imperative that there are sufficient targets and nontargets allowing for participants to make mistakes. As a consequence of the lower number of targets and especially of nontargets in the Go/No-Go task used in this study, we were less capable of capturing accuracy. Therefore, it was not possible to reliably calculate *d*′ in this study, but future studies using Go/No-Go tasks of longer duration should consider including *d*′.

Strengths of this study include the well-matched groups who were free of many factors that could influence Go/No-Go performance, such as poor sleep quality, certain types of medication, and fatigue associated with long work hours, and the inclusion of health care workers who do not have a medical profession. However, no information on symptoms of depression, anxiety, or burnout was available in this study. Furthermore, neuroimaging was not collected, which inhibits a better understanding of the neural correlates of the Go/No-Go task in this sample. Due to the short time of the test and the nonjittered stimulus presentation time, it cannot be ruled out that some participants at some point showed automated responses. However, as all participants completed the test for the first time, automated responses are less likely. Because most participants had either less than 6 or more than 36 hours of exposure time to COVID-19, it was not possible to perform an ordinal regression analysis to evaluate the effects of exposure time in the other groups. Therefore, we opted for performing a MANCOVA. To exclude the effect of fatigue on performance, we excluded those who participated after their shift. However, future studies could adopt a pre- and postshift design, in order to address the question about fatigue in a better way.

In conclusion, we demonstrated that hospital employees with substantial exposure to COVID-19 have a profile of reaction time performance that fluctuates more, i.e., is less stable, as compared to their counterparts with minimal exposure. However, there were no significant differences in test accuracy or in reaction time itself. Whether the variability in reaction times in the substantially exposed group is related to a greater risk of an accidental SARS-CoV-2 infection, other work complications, or greater cognitive or psychological burden needs to be determined in future studies. Future research should, furthermore, focus on the interaction between psychological distress, well-being, fatigue, and attentional functioning in this group ultimately, to be able to better assist those in need of (psychological) counseling.

## Figures and Tables

**Table 1:** Group characteristics of all included participants.

	Minimal exposure to COVID-19	Substantial exposure to COVID-19	*P* value
*N*	44	80	-
Age (years)	39.61 ± 10.68	42.71 ± 10.02	0.110
Sex (male/female; % male)	15/29 (34.1)	28/52 (35.0)	0.999
Education level			0.014
Elementary level (%)	0 (0.0)	1 (1.3)	
High school level (%)	0 (0.0)	10 (12.5)	
Bachelor or higher (%)	44 (100.0)	69 (86.3)	
Exposure time to COVID-19 (%)			-
5 hours or less	28 (63.6)	-	
6 to 11 hours	10 (22.7)	-	
12 to 23 hours	6 (13.6)	-	
24–35 hours	-	16 (20.0)	
36–47 hours	-	48 (60.0)	
48 or more hours	-	16 (20.0)	
Self-reported poor sleep quality (last 2 weeks)			0.639
Never	7 (15.9)	19 (23.8)	
Less than a week	14 (31.8)	26 (32.5)	
More than a week	11 (25.0)	20 (25.0)	
Almost all days	12 (27.3)	15 (18.8)	
Previous COVID-19 infection (%)	4 (9.1)	18 (22.5)	0.085
Profession^a^			0.260
Medical/paramedical (%)	28 (63.6)	42 (52.5)	
Other (%)	16 (36.6)	38 (47.5)	
*Attentional performance*			Cohen's *δ*
Reaction time (ms)	371.33 ± 35.44	389.63 ± 49.08	-0.41
Variability of reaction times (ms)	64.62 ± 16.19	79.53 ± 31.98	-0.55
Coefficient of variability	0.17 ± 0.04	0.20 ± 0.06	-0.56
Commission errors (min–max)	2 (0–10)	3 (0–10)	-0.29
Omission errors (min–max)	0 (0–21)	1 (0–31)	-0.27

Data are presented as mean with standard deviation, absolute numbers with minimum and maximum, or as absolute numbers with percentages between parentheses. ^a^Medical and paramedical professions included medical doctors and medical residents, nurses, physiotherapists, and psychologists. Other professions included nutritionists, pharmacists, engineers, and administrative, security, and laboratory employees.

**Table 2:** Group characteristics of the matched groups.

	Minimal exposure to COVID-19	Substantial exposure to COVID-19	*P* value
*N*	32	32	-
Age (years)	39.06 ± 9.47	40.84 ± 8.47	0.431
Sex (male/female; % male)	9/23 (28.1)	8/24 (25.0)	0.999
Education level			0.113
Elementary level (%)	0 (0.0)	1 (3.1)	
High school level (%)	0 (0.0)	3 (9.4)	
Bachelor or higher (%)	32 (100.0)	28 (87.5)	
Exposure time to COVID-19 (%)			-
5 hours or less	22 (68.8)	-	
6 to 11 hours	7 (21.9)	-	
12 to 23 hours	3 (9.4)	-	
24–35 hours	-	8 (25.0)	
36–47 hours	-	16 (50.0)	
48 or more hours	-	8 (25.0)	
Self-reported poor sleep quality (last 2 weeks)			0.910
Never	7 (21.9)	9 (14.1)	
Less than a week	12 (37.5)	11 (34.4)	
More than a week	3 (9.4)	4 (12.5)	
Almost all days	10 (31.3)	8 (25.0)	
Previous COVID-19 infection (%)	4 (12.5)	10 (31.1)	0.129
Profession^a^			0.430
Medical/paramedical (%)	19 (59.4)	23 (71.9)	
Other (%)	13 (40.6)	9 (28.1)	
*Attentional performance*			Cohen's *δ*
Reaction time (ms)	370.61 ± 35.88	387.57 ± 46.87	-0.41
Variability of reaction time (ms)	63.44 ± 14.16	82.10 ± 29.99	-0.81
Coefficient of variability	0.171 ± 0.035	0.212 ± 0.064	-0.81
Omission errors (min-max)	0 (0–21)	0.50 (0–31)	-0.22
Commission errors (min-max)	2 (0–10)	3 (0–10)	-0.36

Data are presented as mean with standard deviation, as absolute numbers with percentages between parentheses, or as absolute numbers with the minimum and maximum values. ^a^Medical and paramedical professions included medical doctors and medical residents, nurses, physiotherapists, and psychologists. Other professions included nutritionists, pharmacists, engineers, and administrative, security, and laboratory employees.

## Data Availability

Data is available from the corresponding author upon reasonable request.
